# The Quality Management Ecosystem in Cell Therapy in Catalonia (Spain): An Opportunity for Integrating Standards and Streamlining Quality Compliance

**DOI:** 10.3390/cells11132112

**Published:** 2022-07-05

**Authors:** Joaquim Vives, Maria Glòria Sòria, Eoin McGrath, Mara Magri

**Affiliations:** 1 Banc de Sang i Teixits, Edifici Dr. Frederic Duran i Jordà, Passeig Taulat, 116, 08005 Barcelona, Spain; mgsoria@bst.cat; 2 Musculoskeletal Tissue Engineering Group, Vall d’Hebron Research Institute (VHIR), Universitat Autònoma de Barcelona, Passeig de la Vall d’Hebron 129-139, 08035 Barcelona, Spain; 3 Departament de Medicina, Universitat Autònoma de Barcelona, Passeig de la Vall d’Hebron 129-139, 08035 Barcelona, Spain; 4 International Council for Commonality in Blood Banking Automation (ICCBBA), P.O. Box 11309, San Bernardino, CA 92423-1309, USA; emmcgrath@hotmail.com; 5 ASST Papa Giovanni XXIII, Piazza OMS, 1, 24127 Bergamo, Italy

**Keywords:** cell therapy, quality standards, quality compliance, JACIE, GMP, ISO9001, FACT, NetCord, AABB, integrated management system

## Abstract

Cell therapies are required to meet with compulsory regulations that co-exist with other optional standards and guidelines that together compose a complex quality management system. Indeed, reliable insights on the mechanisms of action and safety of novel cell-based therapies require adherence to solid quality management structures in all steps of the value chain, from early research and tissue procurement to clinical trials and biovigilance, thus guaranteeing reproducibility and solid foundations for better science and improved clinical practice. Herein we present the concept of the quality ecosystem as a tool to understand and assist all stakeholders involved in developing and structuring the integration of standards as novel developments are taking place. We conclude that the various quality management initiatives can all be thought about under the umbrella of an ecosystem.

## 1. Introduction

Today’s cell therapies are not restricted to blood transfusion and transplantation of hematopoietic progenitor stem cells only but also encompass sophisticated gene-edited cell-based products such as chimeric antigen receptor (CAR)-T cells or tissue engineering products such as Holoclar [1]. Although essentially different, these products co-exist and comply with specific quality and regulatory requirements that overlap to some extent [2]. In the field of bone marrow transplantation, several standard-setting and accreditation bodies, such as JACIE [the Joint Accreditation Committee of the International Society for Cell and Gene Therapy (ISCT)-European Society for Blood and Marrow Transplantation (EBMT)], the Foundation for the Accreditation of Cellular Therapy (FACT) and the American Association of Blood Banks (AABB), have brought a high degree of harmonization over the last 20 years and more recently are applying many of the well-established quality management and procedural concepts to the new generation of cell-based medicines. 

Through the continuous improvement of the processes involved in the development and production of cell-based therapies, quality standards have evolved and adapted to scientific progress, increasing product consistency, safety and efficacy. Such evolution over time has implied the creation of standards where they did not exist (some of them of voluntary application that, in some cases, preceded the mandatory application of legal rules), thereby covering the diversity of products and their indications as well as their regional governance.

Acknowledging that the human application of tissues and cells is an expanding medical field that offers important opportunities for the treatment of disease but also implies significant risks, regulatory authorities in the EU and US have aimed to establish high quality and safety standards for tissues and cells used in human applications [3]. In the EU, a series of directives were issued describing how human tissues and cells must be collected and processed with a separate legal framework that includes the cells used in the development of so-called advanced therapy medicinal products (ATMP) [4]. 

## 2. Quality Standards in Cell Therapy 

The first standards specific to cell therapy emerged in the US in the mid-1990s through FACT and AABB initiatives. JACIE followed soon afterwards in Europe, whereby the FACT model was adopted with some adaptation to the European context. Elsewhere, the blood sector was increasingly regulated following a number of high-profile crises in Ireland and France, among others. 

Importantly, existing standards have incorporated novel cell-based therapies into their scope. For instance, FACT and JACIE standards include immune effector cells, while AABB offers accreditation of the processing, storage and distribution of ex vivo expanded multipotent mesenchymal stromal cells. Cross-fertilization between standards may have led to the incorporation of certain aspects from stricter standards, e.g., JACIE incorporating risk-based analysis from current good manufacturing practices (GMP) standards. From a wider perspective, this situation suggests a landscape of quality management standards both individually as well as organized into systems of standards covering many different types of cell-based products, their sources and manufacturing processes, sometimes with overlaps between them and clear common objectives of donor and patient safety as well as product efficacy. 

## 3. The Concept of the Ecosystem of Quality Standards 

Looking at the rich field of quality standards, we might wonder how it came to be so. To address this question we have taken an ecological perspective. Briefly, ecological succession is a series of progressive changes in the species that make up a community over time, and it often involves a progression from low species diversity to high species diversity. Primary succession occurs when bare rock is exposed, for instance, after a volcano eruption, providing a habitat that can be colonized for the first time. In the next phase, weathering is required to break down the substrate enough for the establishment of pioneer species such as lichens [5]. In the final stage of primary succession, a climax ecosystem is reached in which the species composition remains relatively stable until a disturbance that alters the equilibrium occurs. Secondary succession follows until a new climax is attained.

The term “ecosystem” refers to an evolving concept that was first formulated in the 1930’s by botanists A.R. Claphan and A.G. Tansley on concepts related to ecology [6]. Since then, its use has reached fields as diverse as education, recreational opportunities, production systems, software development and innovation to explain interactions between “communities” and their “neighbors”. According to Frederick E. Smith, an ecosystem is “a functional unit with recognizable boundaries and an internal homogeneity” [7]. Based on parallels in ecology, we propose the quality ecosystem (QE) to be defined as “a unit comprising a defined field of interest (herein, cell therapy) in which a range of quality standards apply and their regulatory, medical and social environment, at any scale (institutional, regional, national, continental or global), which are continuously influenced by scientific progress, patient needs, political rulings, ethics and economical pressure in an interactive open system”. 

The behavior of the QE with time surpasses continuous improvement by the evolution and selection of specific standards or parts thereof that better fulfill the requirements in specific developments or the manufacture of cell-based products. A clear selective evolutionary pressure in current quality management ecosystems is the medicalization of traditional cell-based products (i.e., blood and hematopoietic progenitor stem cells) in accordance with new laws, implying the need to implement GMP-like requirements (e.g., the Good Practice Guidelines for blood establishments by the European Directorate for the Quality of Medicines and Healthcare; EDQM) with the effect of pushing other standards to incorporate risk-based management and stricter documented qualification of equipment, people and processes not only for the handling of cells but also tissues (e.g., EuroGTP II) [8,9,10]. 

## 4. The Quality Management Ecosystem of Cellular Immunotherapy 

Hematopoietic stem cell transplantation (HSCT) is currently used in the treatment of a variety of life-threatening conditions, including hematologic malignancies and autoimmune, inflammatory and genetic disorders [11]. Given that allogeneic HSCT entails potentially serious complications, such as graft vs. host disease (GvHD) or susceptibility to opportunistic infections, adoptive cellular immunotherapy and/or manipulation of the graft offer a means to fight against residual cancer as well as other transplant-related complications. In this sense, donor lymphocyte infusion (DLI) has been traditionally used as an effective immunotherapeutic approach (offering a graft-versus-leukemia, GvL, effect) with significant activity in the treatment and prevention of relapse after allogeneic HSCT [12]. However, DLI is associated with significant toxicity mainly due to unwanted GvHD and does not produce durable responses in aggressive malignancies (e.g., B-cell acute lymphoblastic leukemia, B-ALL). In this context, the irruption of revolutionary CAR T-cell therapies for the treatment of hematological malignancies (including acute lymphoblastic leukemia, chronic lymphocytic leukemia, lymphoma and multiple myeloma) has prompted to the evaluation of the efficacy of this novel approach over traditional HSCT, leading to the promising, safe and potentially effective action of anti-CD19 CAR T-cell therapy for relapsed B-ALL after allo-HSCT, which may be superior to DLI [13]. Despite outstanding the initial responses observed with CD19 CAR T cells for younger patients with B-cell acute lymphoblastic leukemia, historical data show more than half of patients experience disease relapse within 4 to 5 years, so current research is focused on whether HSCT after CAR T-cell therapy may have a positive effect on treatment durability, and indeed, CD19-directed CAR T-cell therapy before HSCT was shown to lead to more durable remissions [14], with important implications for future practice guidelines. 

We believe that strict adherence to quality management standards in all aspects of cell therapy development, production and human testing as well as donor selection and patient follow-up is crucial to produce high quality data that contribute to shedding new light on the mechanisms of action involved. This is particularly relevant in novel cellular immunotherapies (i.e., tumor infiltrating lymphocytes, TIL; virus-specific T cells, VST; CAR-T) not only to understand molecular mechanisms and enhance efficacy (i.e., those mediated by CAR-T-based therapies) but also to improve their safety profiles. Remarkable efforts are currently being made to develop innovative strategies to control CAR-T activity, for instance, with the aim of making them safer by mitigating toxicities while addressing the unique biology of diverse hematological and solid malignancies [15]. 

Furthermore, real world data may hold the potential to provide massive, clinically relevant datasets that would further contribute to generating novel insights into the effectiveness of innovative cell- and gene-based therapies that otherwise had to be tested in underpowered cohorts of patients (much smaller than traditional small-molecule drugs or biologics due to the complexity of handling living medicinal products). Indeed, the use of real world data is gradually growing, moving beyond drug development to population health. To harness the potential of this approach, high-quality datasets are urgently needed, entailing adherence to standards on data (i.e., entry, equity) and tailored quality management assurance systems [16]. 

## 5. Case Study 

To explore the temporal evolution of QE and analyze the succession of quality standards in the field of transfusion and cell therapy, we used Catalonia, Spain as the geographic and temporal boundaries of this ecosystem under study, from the establishment of the first blood bank up to today. 

### 5.1. Pioneers and Primary Succession 

Even though blood circulation was already known in the 16th century, and early transfusions were attempted although unsuccessfully, it was Landsteiner’s discovery of the ABO blood group in 1901 that launched transfusion as a therapy. The discoveries of citrate as an anticoagulant and glucose as a preservative in 1914 were also much needed elements for the storage of blood [17]. After the experience of transfusion during the First World War, the substrate was ready for the appearance of blood banks.

At the start of the Spanish Civil War, in 1936, Frederic Duran i Jordà, a young medical doctor, was put in charge of transfusion in Barcelona. He designed and managed what can be considered the first modern blood bank: based on altruistic donations, with standardized procedures and strict quality controls. He came up with innovative solutions to preserve and transport refrigerated blood to the battle front, 300 km away. In two and a half years, the Barcelona blood bank obtained and transfused 9000 L of blood. While in exile in Great Britain, Duran published his procedure and shared his experience to help establish other banks during the Second World War [18].

Could we regard this procedure as the first pioneer species of quality standards colonizing a bare rock? Pioneer species contribute to soil formation where other less hardy species can later grow. Having prepared the substrate, the subsequent colonization of other lichens, mosses, herbs and shrubs is much faster.

The following decades saw the discovery of other blood groups, compatibility tests, new blood preservatives, plastic bags, etc. Being conscious of the great benefits but also the great risks of transfusion, blood bank professionals soon associated to share experiences. The International Society of Hematology (ISH) was established in 1946, and the AABB followed in 1947. In our particular ecosystem, the AEHH (Asociación Española de Hematología y Hemoterapia) was founded in 1959. Actively participating in the AEHH, hospital-based and private blood banks flourished in Barcelona, as was happening elsewhere at the time. The AEHH soon set up quality standards for transfusion and blood banking and launched the PABAS (Programa de Acreditación de Bancos de Sangre) voluntary accreditation scheme in 1973. While legal regulation of blood banks was established in 1975 in Spain, it was mainly administrative, without technical and quality aspects and, therefore, PABAS was a decisive instrument to ensure quality in transfusion medicine and soon several blood banks were accredited (Table 1). Meanwhile, new scientific advances were taking place in the field of hematology. In 1976 in Barcelona, Ciril Rozman performed the first documented allogenic bone marrow transplant in Spain following the example of the Seattle group. Similar to the food chain in which secondary producers feed on primary producers in a biological ecosystem, hematopoietic progenitor transplant units drew on the experience of blood banks, such as with cross-matching techniques or donor registers, and rapidly developed in several hospitals. A climax ecosystem had been established, but it would soon be disturbed. 

### 5.2. Secondary Succession

Ecologists call secondary succession a re-colonization of a previously occupied area following a disturbance that destroys much or all of its community, a classical example being a forest cleared by wildfire. Similarly, our climax ecosystem was shaken in the 1980s and 90s by the public awareness of transfusion-transmitted cases of acquired immunodeficiency syndrome and hepatitis. Today, blood transfusion is an extremely safe procedure, but it has not been always like this. The increasing use of blood and blood components had a parallel increase in transfusion-associated hepatitis infections and became widely reported in the media. Importantly, the hepatitis B virus had been discovered in 1967, but the human immunodeficiency virus (HIV) was only discovered in 1984, and the hepatitis C virus (HCV) in 1989 [19,20]. Like a fire that destroys but also renews an ecosystem through the species that survive, the Spanish government, faced with the severe risk of infection, introduced stricter legislation for blood banks in 1985. Remunerated donations were forbidden, voluntary donor associations were promoted, a network of community blood banks was designed and technical and quality requirements were legally established, including for donor selection criteria and laboratory tests. Mandatory quality standards were inspired by the existing international voluntary accreditation schemes, thus giving rise to a sort of symbiosis between the two sets of requirements.

In 1986, Spain joined the European Union (EU). One of the innumerable consequences of this event was the gradual adaptation to European standards, including the transposition of EU directives into Spanish law. The following years saw several new laws aiming at adapting testing to the state of the art by mandatory HCV and HIV screening. A significant transformation took place among blood banks in our ecosystem as a result.

Another significant event in the secondary succession of this ecosystem was the well-publicized case of Josep Carreras, a famous Catalan opera singer, who following transplantation recovered from leukemia and soon afterwards, in 1988, established the José Carreras Foundation (www.fcarreras.org, accessed on 1 April 2022), which in turn led to the creation of REDMO (Registro de Donantes de Médula Ósea), the Spanish national unrelated donor register, and through charity fund-raising also greatly contributed to scientific research in cell therapy. 

In 1995, Banc de Sang i Teixits (BST; Barcelona, Spain) was created as a public agency of the Catalan Department of Health, with the mission to guarantee the supply and proper use of human blood and tissue in Catalonia and to be the reference center in the field of immunodiagnostics and the development of advanced cell therapies (www.bancsang.net, accessed on 1 April 2022). BST was born through the cooperation and fusion of the main hospital blood banks in Catalonia, thus preserving a network structure deeply rooted in the territory. The unique structure of BST, with a GMP-compliant central processing unit and clinical units in the major Catalan hospitals, was key in the development of the so called “vein-to-vein model”, taking pride in controlling the quality of the whole process, from the vein of the donor to the vein of the recipient. Donation centers are centrally managed through the BST network, all donated products are processed in BST facilities, and almost all transfusions are performed in hospitals under BST supervision. 

Today, BST hosts a blood bank, a cord blood bank, a tissue bank, a human milk bank, a hematopoietic stem cell processing unit and an advanced cell therapy unit as well as ongoing research and all the necessary support processes: donation promotion, logistics, testing laboratories, quality, etc. The coexistence of knowledge, talent and technology cradles the advancement of new cell therapies as a natural evolution of blood transfusion. Indeed, one of the main reasons for the creation of BST was to facilitate the implementation of high quality standards beyond the mandatory ones. Fulfilling this vision, BST realized that implementation of a general quality system was vital and decided to apply ISO 9001 principles, which is a general standard that has experienced a rapid expansion throughout Europe due to its capacity to be implemented in any kind of organization, from industry or services to the primary sector. With the same generalist strategy, ISO 14001, OHSAS 18001 (Occupational Health and Safety Management System that is currently replaced by ISO 45001, so organizations previously certified under OHSAS 18001 had to migrate to ISO 45001 before 12 March 2021) and EFQM (European Foundation Quality Model) have also seen significant uptake in Europe (Table 2). These four standards may be applied in any organization without overlapping, so they not compete with each other. Instead, each standard has found its own thematic niche: ISO9001 deals with quality management, ISO14001 with environmental management, OHSAS/ISO 45001 with safety management and EFQM with corporate management. On the other hand, BST holds the following specific accreditations: CAT (formerly PABAS) for blood transfusion; CAT and NETCORDFACT for cord blood banking; FACT-JACIE for HSCT; and EFI for histocompatibility testing (Table 2). These standards are very specific and cover donation, collection, testing, processing, storage, distribution and transfusion or transplantation. The sustained relevance of any of these standards relies on finding a “secluded niche”, since they can only be implemented in a limited number of organizations around the world and are rarely subject to competition. If they occasionally do encounter competition, the standards tend to converge, fuse or harmonize, leaving eventually only one standard per field at the national or international level. We have to bear in mind that these specific voluntary standards are developed through consensus by professionals collaborating via congresses and expert working parties. More advanced members push for higher standards and others eventually follow. Hence, standards are always striving for excellence. 

They are revised continually to adapt to technological improvements or new legal requirements, and new editions are introduced on a regular basis. 

Regarding mandatory requirements, although transfusion and transplantation are clearly cell therapies, they have so far not been categorized as drugs. However, extensively manipulated cells or cells used for another function are indeed considered pharmaceutical drugs and must comply with GMP according to the US Food and Drug administration (FDA) and European Medicines Agency (EMA), among other regulators. GMP regulation seeks to ensure the identity and potency of purity of drug products through extensive control of manufacturing processes and facilities, and quality management systems are the key to achieve this control. Adapting GMP, designed for the manufacturing of chemical drugs in pharmaceutical settings, to cellular products has been challenging, but BST has successfully maintained compliance with GMP since 2011, upon inspection by the Spanish 284 Regulatory Authority (Agencia Españolas de Medicamentos y Productos Sanitarios, AEMPS) (2). Certification for GLP was successfully achieved to perform preclinical studies required in the development of ATMP but the lack of selective pressure from the regulatory authorities and the versatility of GMP (covering both pharmaceutical production and validation of processes in the technology transfer arena) led to discontinuation of certification in 2018.

ATMPs are complex and diverse products with some degree of variability due to the use of biological materials, complex manipulation steps, limited batch sizes and the inherent variability in the starting material [21]. ATMPs are currently at the frontier of scientific innovation and are often initially developed in academic or hospital settings rather than large pharmaceutical companies [2,22]. Considering all this, GMP requirements currently applicable to ATMPs take a risk-based approach [10]. Therefore, the manufacturer can design the measures needed for compliance with GMP according to the specific risks of the product and the manufacturing process. However, to substantiate this approach, the quality system required has to be especially robust, going beyond ISO9001. Indeed, most advanced cell therapy units have strived to achieve the required level, either by creating specific GMP quality units or by further developing the existing quality management system. BST has opted for avoiding duplication and effort waste. A detailed analysis of the quality systems required by CAT, EFI, ISO, FACT-NETCORD, JACIE or GMP shows a coincidence of spirit, beyond the actual requirements. Moreover, time has shown us that voluntary standards tend to converge and grow stricter. Similarly, mandatory requirements behave in the same way. Indeed, the European Directorate for the Quality of Medicines & HealthCare (EDQM) and the Commission of the European Union also established stricter guidelines for blood transfusion and for tissues and cells for human application. 

Directive (EU) 2016/1214, published in July 2016, requests EU member states to ensure that blood establishments comply with the Good Practice Guidelines (GPG) for their quality system by 15 February 2018. GPG requires a quality system that encompasses quality management, quality assurance, continuous quality improvement, personnel, premises and equipment, documentation, collection, testing and processing, storage, distribution, quality control, external and internal auditing, contract management, non-conformance, self-inspection, a formal change control system and risk analysis, among other aspects. 

The EDQM published the guide to the quality and safety of tissues and cells for human application first in 2013, and it is now in its 4th edition. It collates the most up-to-date information to provide professionals with technical guidance on ensuring the quality and safety of human tissues and cells applied to patients. This guide includes recommendations considered the “minimum standards” that align with the principles set out in the various relevant European Union (EU) directives. However, the guide goes further by providing additional technical advice based on GMP guidelines and on best practices consistent with current scientific knowledge. It also refers to recent developments that may be reflected in future updates of EU legislation. 

BST has created a quality commission formed by key members of all product divisions and support processes with the aim of designing a unique central quality system that covers all standards followed by BST, either voluntary or mandatory. By adopting the strictest guideline in every aspect, quality manual and general procedures are being prepared, including a unique quality information system. More advanced divisions are helping others to achieve the required levels. It is envisaged that this approach will also allow simplification of the internal audit calendar since all applicable requirements will be audited at the same visit. 

We are again approaching a climax ecosystem, one where cooperation between blood banking and cell therapy is stronger than ever to bring to patients a new generation of innovative, personalized cell-based therapies. 

## 6. Outlook

Our concept of quality ecosystems aspires to be operationally useful and anticipate situations such as trends towards global governance, reduction in the number of standards (by merges and/or eliminating overlaps and redundancies), redefinition of the scope of specific standards (i.e., to adapt to scientific progress) or to highlight potential integration of standards in manageable systems in institutions producing different types of cell-based products. Taken together, the key contribution of implementing and developing mature quality management environments is to guarantee the reproducibility of experimental data and provide solid foundations for better science and improved clinical practice. 

## Figures and Tables

**Table 1:** Applicable laws and guidelines in the evolution of cell therapy quality management systems in Spain.

Rules	Topic	Country	Year
“Decreto 1574/1975 de 28 de junio, por el que se regula la hemodonación y los Bancos de Sangre”	Blood banks	Spain	1975
“Orden de 4 de diciembre de 1985 de desarrollo del Real Decreto 1945/1985, de 9 de octubre, por la que se regula la hemodonación y los Bancos de Sangre, determinando, con carácter general, requisitos técnicos y condiciones mínimas en la materia”	Blood banks, hemodonation	Spain	1985
“Real Deecreto 1945/1985, de 9 de octubre por el que se regula la hemodonación y los Bancos de Sangre”	Blood banks, hemodonation	Spain	1985
“Orden de 18 de febrero de 1987 sobre pruebas de detección anti-VIH en las donaciones de sangre”	HIV testing	Spain	1987
“Orden de 3 de octubre de 1990 sobre pruebas de detección de anticuerpos del virus de la hepatitis C (anti-VHC) en las donaciones de sangre”	HCV testing	Spain	1990
“Real Decreto 1854/1993, de 22 de octubre, por el que se determina con carácter general los requisitos técnicos y condiciones mínimas de la hemodonación y bancos de sangre”	Blood banks, hemodonation	Spain	1993
Regulation (EC) No 1394/2007 on advanced therapy medicinal products and amending Directive 2001/83/EC and Regulation 372 (EC) No 726/2004	ATMP	EU	2007
European Directorate for the Quality of Medicines & HealthCare (EDQM). Guide to the quality and safety of tissues and cells for human application 4th Edition.	Cells, Tissues	EU	2019

**Table 2:** Quality ecosystem at Banc de Sang i Teixits.

General	Blood	Cord Blood	HSCT	Histocompatibility Testing	ATMP	Standard	Issuer	Reference	Year of First Certification
X						ISO9001	ISO	Quality management systems	1999
X						ISO14001	ISO	Environmental management systems	2013
X						OHSAS18001	BSI	Occupational health and safety management systems	2015 *
X						EFQM	EFQM	Recognition for excellence in Quality Management	2010
	X					CAT	FCAT	Standards in hemotherapy	2005
	X					GPG	EDQM	The Guide to the preparation, use and quality assurance of blood components	**
		X				FACT-NETCORD	FACT	International Standards for Cord Blood Collection, Banking, and Release for Administration	2005
		X				CAT	FCAT	CAT standards for the procurement, processing, storage and distribution of cord blood	2011
			X			FACT-JACIE	JACIE	FACT-JACIE International Standards for Hematopoietic Cellular Therapy. Product collection, processing and administration	2015
				X		EFI	EFI	Standards for histocompatibility	2002
					X	GLP	AEMPS	Principles on Good Laboratory Practice. Series on Principles of Good Laboratory Practice and Compliance Monitoring	2014 ***
					X	GMP	AEMPS	The Rules Governing Medicinal Products in the European Union Volume 4 Good Manufacturing Practice. Guidelines on Good Manufacturing Practice Specific to Advanced Therapy Medicinal Products	2010

(*) Expired in 2021, not transitioned to ISO 45001; (**) interim audits performed but no official certification exists to date; (***) certification not renewed in 2018.

## Data Availability

Not applicable.

## Abbreviations

AABB	American Association of Blood Banks
AEHH	Asociación Española de Hematología y Hemoterapia
AEMPS	Agencia Española de Medicamentos y Productos Sanitarios
ATMP	Advanced Therapy Medicinal Products
B-ALL	B-cell Acute Lymphoblastic Leukemia
BSI	British Standards Institute
BST	Banc de Sang i Teixits
CAR-T	Chimeric Antigen Receptor T cells
CAT	Calidad en Transfusión Sanguínea
EBMT	European Society for Blood and Marrow Transplantation
EDQM	European Directorate for the Quality of Medicines & HealthCare
EFI	European Federation for Immunogenetics
EFQM	European Foundation Quality Model
EMA	European Medicines Agency
EU	European Union
EuroGTP II	European Good Tissue and Cell Therapies II
FACT	Foundation for the Accreditation of Cellular Therapy
FCAT	Fundación para la Calidad en la Transfusión Sanguinea, Terapia Celular y Tisular
FDA	Food and Drug Administration
GLP	Good Laboratory Practice
GMP	Good Manufacturing Practice
GPG	Good Practice Guidelines
GvHD	Graft versus Host Disease
GvL	Graft versus Leukemia
HBV	Hepatitis B Virus
HSCT	Hematopoietic Stem Cell Therapy
HCV	Hepatitis C Virus
HIV	Human Immunodeficiency Virus
ISH	International Society of Hematology
ISO	International Organization for Standardization
JACIE	Joint Accreditation Committee International Society for Gene and Cell Therapy (ISCT)-European Society for Blood and Marrow Transplantation (EBMT)
OECD	Organization for Economic Cooperation and Development
OHSAS	Occupational Health and Safety Management System
PABAS	Programa de Acreditación de Bancos de Sangre
QE	Quality Ecosystem
REDMO	Registro de Donantes de Médula Ósea
TIL	Tumor Infiltrating Lymphocytes
US	United States of America
VST	Virus-Specific Lymphocytes
